# SARS-COVID-19 transformed the world and urological practice

**DOI:** 10.1590/S1677-5538.IBJU.2020.S1ED1

**Published:** 2020-07-27

**Affiliations:** 1 Universidade do Estado de Rio de Janeiro - Uerj Unidade de Pesquisa Urogenital Rio de JaneiroRJ Brasil Unidade de Pesquisa Urogenital - Universidade do Estado de Rio de Janeiro - Uerj, Rio de Janeiro, RJ, Brasil; 2 Hospital Federal da Lagoa Rio de JaneiroRJ Brasil Serviço de Urologia, Hospital Federal da Lagoa, Rio de Janeiro, RJ, Brasil

SARS-COVID-19 pandemic changed the World and urological practice. The SARS-COVID-19 pandemic has a great impact in all medical fields and because the important changes in Urology in last 3 months and due to the large volume of information and articles received, we chose to carry out this supplement. Despite these extremely difficult times, we are very happy because this supplement marks the beginning of the partnership between the Brazilian Society of Urology (BSU) and the American Confederation of Urology (CAU). Soon the International Brazilian Journal of Urology has everything to become the official information Journal of CAU reinforcing the impact of the International Brazilian Journal of Urology on the American continent.

This Supplement presents original contributions with a lot of interesting papers about SARS-COVID-19 pandemic. The papers came from many different countries such as Brazil, USA, United Kingdom, Belgium, Colombia, Peru, Argentina, Germany, Netherlands, Italy, Spain, Canada, and China. As usual, the editor highlights some of them.

Dr. Lauxman and colleagues from Germany performed in page 6 ( 1 ) a nice explanation about the SARS-CoV-2 and COVID-19 outbreak and they observed that in most severe COVID-19 patients, the D-dimer level is significantly increased showing frequent clotting disorders and microthrombotic formations. This study is on the cover in this number.

Dr. Chen and colleagues from China performed in page 19 ( 2 ) a nice report about the Strategies and Management of Urological Diseases during the COVID-19 Pandemic in China and concluded that the patients seen by urologists are mostly elderly people, who are the frequent population suffering from severe diseases.

Dr. Esperto and colleagues from Italy performed in page 26 ( 3 ) an interesting report about the pandemic impact in Italy and concluded that COVID-19 emergency is a highly dynamic situation and the burden on the healthcare system varies daily according to the geographical region.

Dr. Sanchez and colleagues from Spain performed in page 50 ( 4 ) an important report about prostate cancer assistance during the COVID-19 pandemic and concluded that prostate biopsies should be delayed; androgen deprivation therapy allows us to defer definitive local treatment in many cases of intermediate and high risk prostate cancer and metastatic and castration resistant prostate cancer, combination therapies with abiraterone, apalutamide, darolutamide or enzalutamide could be considered. Chemotherapy, Radium-223 and immunotherapy are discouraged.

Drs. Zequi and Abreu from Brazil and Uruguay performed in page 69 ( 5 ) a nice report about the management of renal cell carcinoma (RCC) during COVID-19 pandemic and shows that in the pandemic COVID-19 times, a tailored risk-based approach must be used for a safe management of RCC, aiming to not compromise the oncological outcomes of the patients.

Dr. Casco and colleagues from Spain performed in page 86 ( 6 ) an important report about the therapeutic and surgical indications of patients with Penile Cancer in COVID-19 era and proposed an action protocol to facilitate decision-making, and concluded that in case of superficial non-invasive disease, topical treatment is effective in absence of lymph node involvement. In selected patients, radiotherapy is an organ-preserving approach with good results. Non-deferrable surgical treatment must be performed by an experienced surgeon and as an outpatient procedure when possible. When indicated, the inguinal lymphadenectomy should not be delayed since it is decisive for patient survival.

Dr. Ibarra and colleagues from Spain Italy and Iran performed in page 104 ( 7 ) a nice review about the impact of the COVID-19 pandemic on sexual behavior in the population from three different countries: Iran, Italy and Spain from each country's perspective and concluded that in the upcoming months and years, we will be able to assess these effects in more detail, but we are sure that COVID-19 will have a negative impact not only in terms of affectivity but also in terms of sexual relationships. The impact of the coronavirus will be very important in the sexual life of the people and we will attend in the next months or years, to some changes in the relationships at all the levels.

Dr. Gonzalez and Ciancio from USA and Spain performed in page 145 ( 8 ) an important report about the risk factors, clinical presentation, therapeutic protocols, and outcomes of kidney transplantation recipients (KTRs) who become infected by SARS-CoV-2 and concluded that the ideal treatment for KTRs with SARS-CoV-2 infection remains unclear, and the answers regarding its optimal management still rely on expert opinion and long-term follow-up is required to better understand the prognosis and sequelae of COVID-19 in KTRs.

Drs. Zampolli and Rodriguez from Brazil and USA performed in page 215 ( 9 ) a nice report about the use of laparoscopy and robotics during the pandemic COVID19 and concluded that modifications of standard practices during minimally invasive surgery such as using lowest intra-abdominal pressures possible, controlled smoke evacuation systems, and minimizing energy device usage are recommended.

The Editor-in-chief expects everyone to enjoy reading of this supplement and for sure better times will come soon around the World.
